# Supplementary material for: Quality evaluation of ground improvement by deep cement mixing piles via ground-penetrating radar
Source: Nat Commun. 2023 Jun 10;14:3448. doi: 10.1038/s41467-023-39236-4 (PMC10257722; doi:10.1038/s41467-023-39236-4)
Supplement: Supplementary file 1 — Supplementary Information [file 41467_2023_39236_MOESM1_ESM.pdf]

Supplementary Information

Quality evaluation of ground improvement by deep cement mixing piles via ground-penetrating radar

Hongyan Shen[1,2*], Xinsheng Li[3], Ruifeng Duan[3,4*], Yong Zhao[5], Jing Zhao[1,2], Han Che[1,2], Guoxin Liu[1,2], Zhijia Xue[6], Changgen Yan[6], Jiwei Liu[6], Chao Jiang[7], Boke Li[3], Hong Chang[3], Jianqiang Gao[6], Yueying Yan[1*]

[1] School of Earth Sciences and Engineering, Xi′an Shiyou University, Xi′an 710065, P.R. China

[2] Shaanxi Key Laboratory of Petroleum Accumulation Geology, Xi′an 710065, P.R. China

[3] Shaanxi Geo-mining Geophysical and Geochemical Exploration Team Co. Ltd., Xi′an 710043, P.R. China

[4] National Engineering Research Center of Offshore Oil and Gas Exploration, Beijing 100028, P.R. China

[5] Shaanxi Land Construction Surveying, Planning and Design Institute Co. Ltd., Shaanxi Land Construction Group, Xi′an 710075, P.R. China

[6] School of Highway, Chang′an University, Xi′an 710064, P.R. China

[7] Gansu Luqiao Highway Investment Co., Ltd., Lanzhou 730030, P.R. China

---

Corresponding authors

E-mail address: shenhongyan@xsyu.edu.cn; 450991137@qq.com; Yanyueying@xsyu.edu.cn

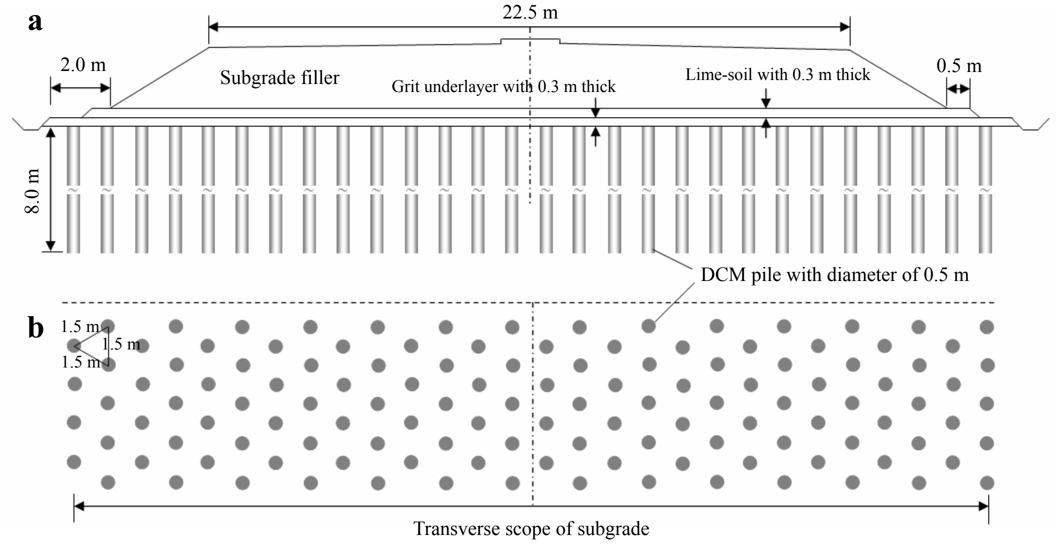

Supplementary Fig. S1 | Schematic diagram of the implementation scheme of subgrade reinforcement by DCM piles. a Vertical section of subgrade. **b** Plan of subgrade (a part).

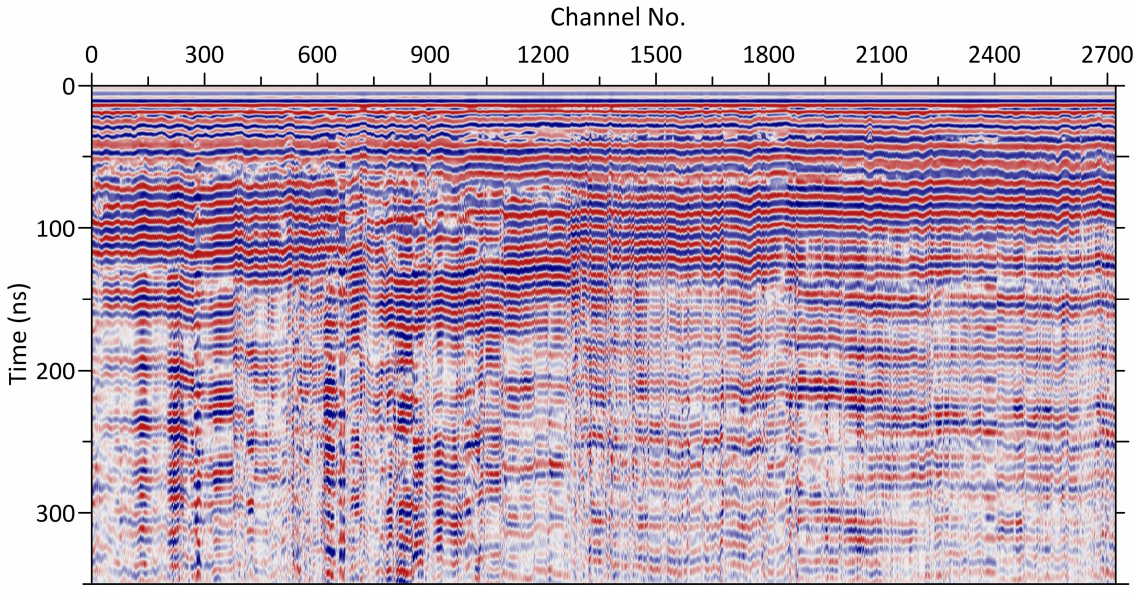

Supplementary Fig. S2 | Original GPR data. Source data are provided with this paper.

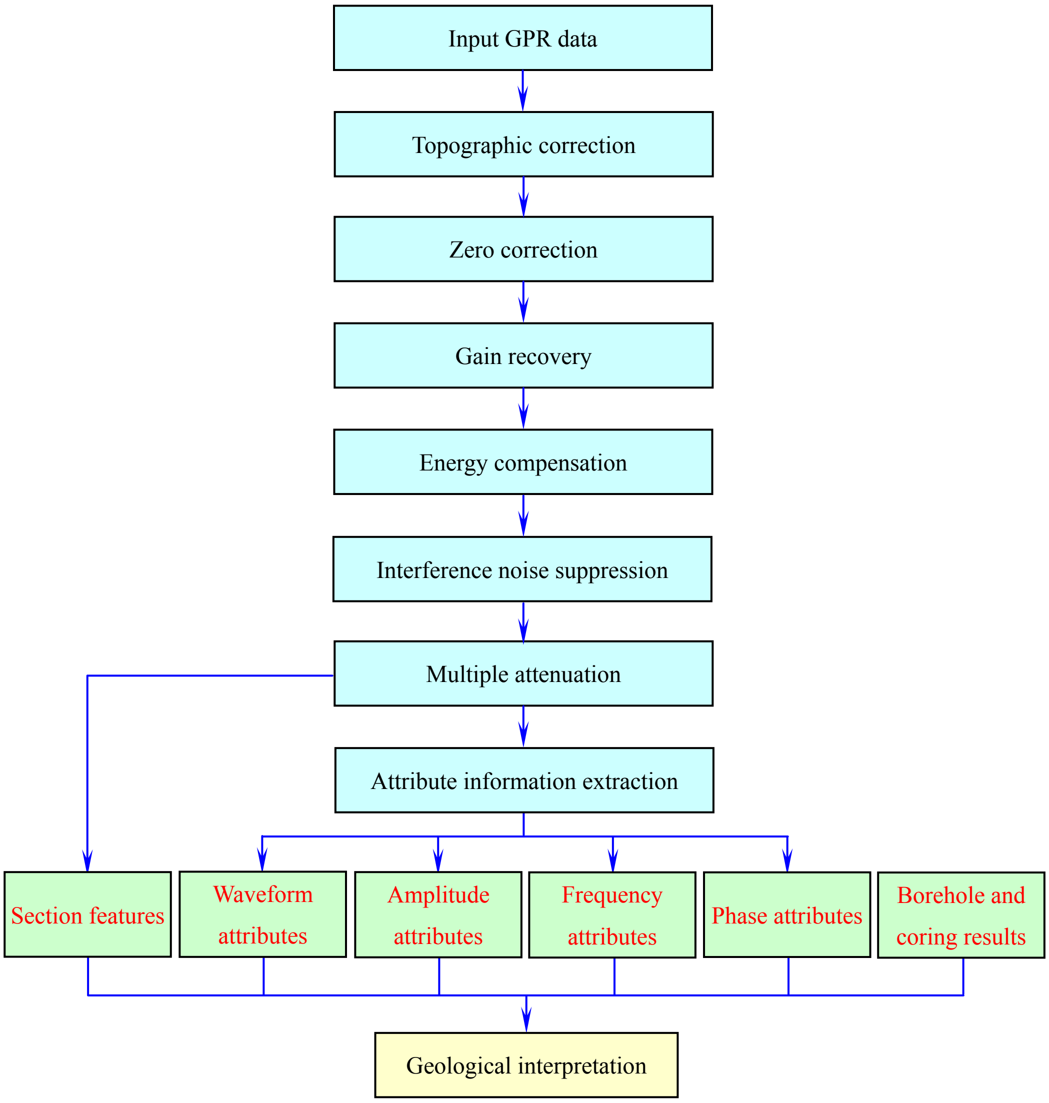

Supplementary Fig. S3 | Flowchart of GPR data processing and geological information interpretation.
