# Supplementary material for: Quality evaluation of ground improvement by deep cement mixing piles via ground-penetrating radar
Source: Nat Commun. 2023 Jun 10;14:3448. doi: 10.1038/s41467-023-39236-4 (PMC10257722; doi:10.1038/s41467-023-39236-4)
Supplement: Supplementary file 3 — Description of Additional Supplementary Files [file 41467_2023_39236_MOESM3_ESM.pdf]

Description of Additional Supplementary Files

File Name: Supplementary Software 1
Description: GPR attribute analysis software and source code.
