# Supplementary material for: Quality evaluation of ground improvement by deep cement mixing piles via ground-penetrating radar
Source: Nat Commun. 2023 Jun 10;14:3448. doi: 10.1038/s41467-023-39236-4 (PMC10257722; doi:10.1038/s41467-023-39236-4)
Supplement: Supplementary file 4 — Supplementary Software 1 [file 41467_2023_39236_MOESM4_ESM.zip › Supplementary Software/GPR_Attribute Software Operation Manual.pdf]

GPR_Attribute Software Operation Manual

Hongyan Shen, Ruifeng Duan, Yueying Yan

Contents

- Overview
- Software Requirements
- Hardware Requirements
- Function
- Installation Guide
- Operating instructions
- Demo

1. Overview

The traditional interpretation of GPR data is based on the variation characteristics of waveform, amplitude and frequency to interpret the geological information carried by GPR section. In fact, the variation characteristics of waveform, amplitude and frequency only reflect one aspect of the geological information carried in GPR data. In the case of obvious underground reflection characteristics, these information can effectively reflect the relationship between GPR response characteristics and geological structure. However, the underground structure and physical properties are complex and variable. When the difference in wave impedance is not significant, the changes in the waveform, amplitude and frequency characteristics of GPR will not be very significant. But it does not indicate that the geological structure or physical properties have not changed. Therefore, relying solely on the waveform, amplitude and frequency characteristics of the GPR section often makes it difficult to accurately reveal the characteristics of underground structures and

the distribution of physical properties. GPR attribute refers to extracting geometric, kinematics, dynamic and statistical characteristics of electromagnetic wave response and propagation from GPR records. GPR attribute analysis is to describe the structure and physical property distribution information of underground targets by extracting descriptive and quantitative GPR attribute characteristics. To this end, we developed a set of GPR attribute analysis software with 4 types of GPR attributes using Visual Fortran 9.0 language. These GPR attributes are particularly suitable for solving geological problems in the fields of engineering and environment.

2. Software Requirements

Windows 7 and above operating systems.

3. Hardware Requirements

The computer processor should be at least 80586DX, with at least 4 G of memory and at least 200 G of hard disk.

4. Function

The GPR attributes include: Arc length of time window (AL), Product of instantaneous amplitude and cosine of instantaneous phase (PIACIP), Slope of instantaneous frequency (SIF) and Slope of reflection strength (SRS).

5. Installation Guide

No installation. The GPR attribute analysis software is an executable program (*.exe).

6. Operating instructions

Open the folder where the GPR_Attribute v1.0 software is located. Double click on the parameter card dialog box GPR_Attribute.par (Figure 1) and modify the relevant parameters. Then save and double-click to execute GPR_Attribute v1.0.exe to perform GPR attribute analysis processing. The parameter descriptions in the

parameter dialog box are shown in Table 1.

```
=======================================================
Function: GPR Attribute Analysis
=======================================================
Input File                     :Line10F1.sgy
Output File                    :Line10F_PF1.sgy
Time Window(ns)                :1.0
Function (1-4)                 :1
=======================================================

Function:
   1--Slope of Reflection Strength (SRS)
   2--Slop of Instantaneous Frequency (SIF)
   3--Product of Instantaneous Amplitude and Cosine of Instantaneous Phase (PIACIP)
   4--Arc length of Time Window (AL)
```

Fig.1 Parameter dialog box of GPR_Attribute

Table 1 GPR_Attribute parameters and their meanings

Parameter	Parameter Meaning
Input File	Enter the original GPR data file. Segy file in microcomputer format without roll head (*. sgy).
Output File	Output GPR attribute data file. Segy file in microcomputer format without roll head (*. sgy)
Time Windows (ns)	Time window length.
Function	Selection switch for GPR attribute analysis.

7. Demo

As an example, a GPR dataset was processed. A total of 2722 channels were collected, with sampling rate of 0.351 ns and sampling length of 359.424 ns. The original GPR data is shown in Figure 2, the SRS attribute extraction results are shown in Figure 3, the SIF attribute extraction results are shown in Figure 4, the PIACIP attribute extraction results are shown in Figure 5, and the AL attribute extraction results are shown in Figure 6. The running time for calculating each GPR attribute results was approximately 5 seconds.

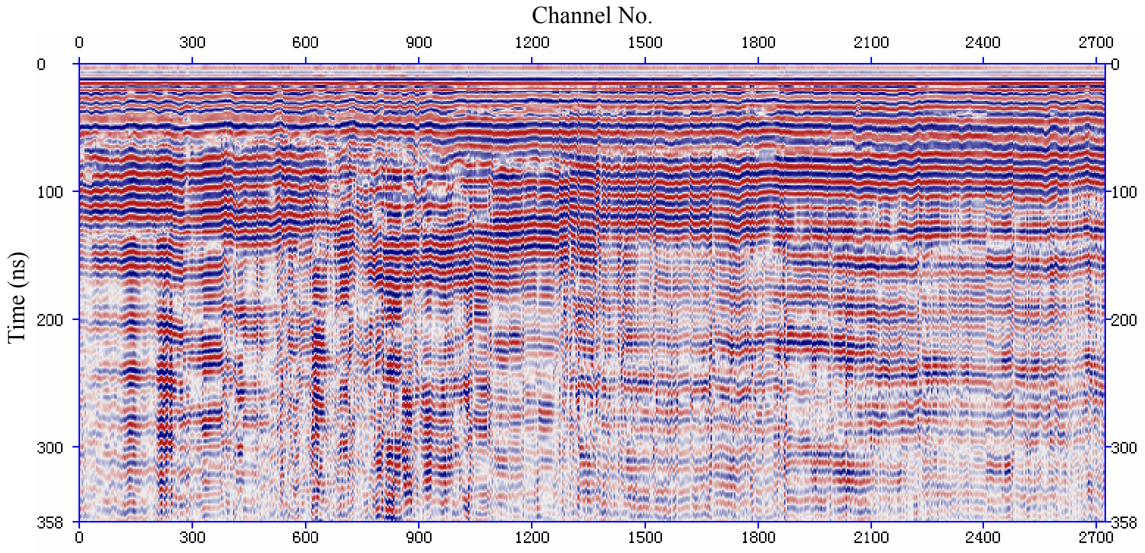

Fig.2 Original GPR data

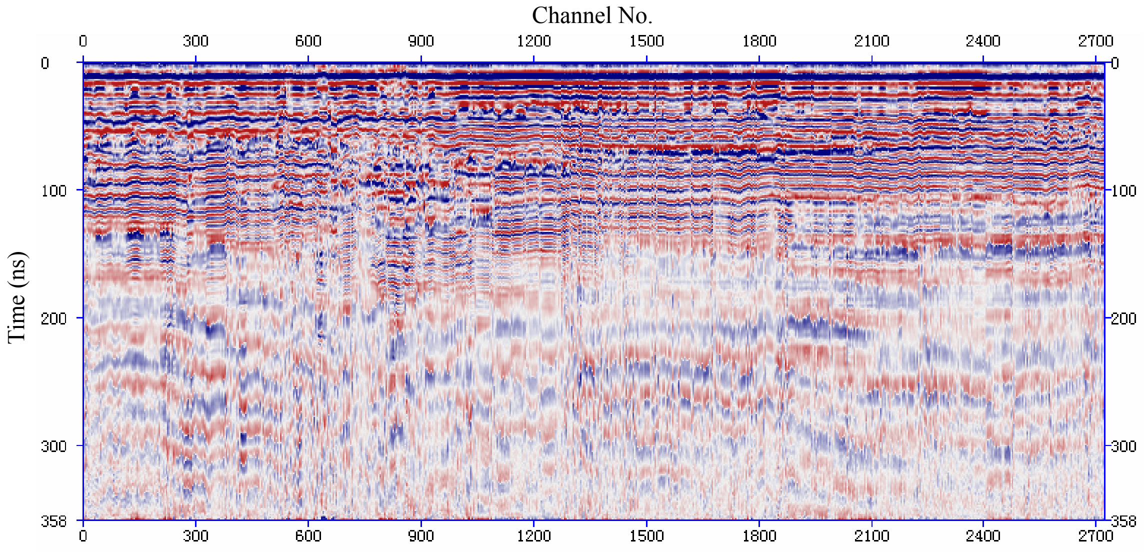

Fig.3 SRS attribute

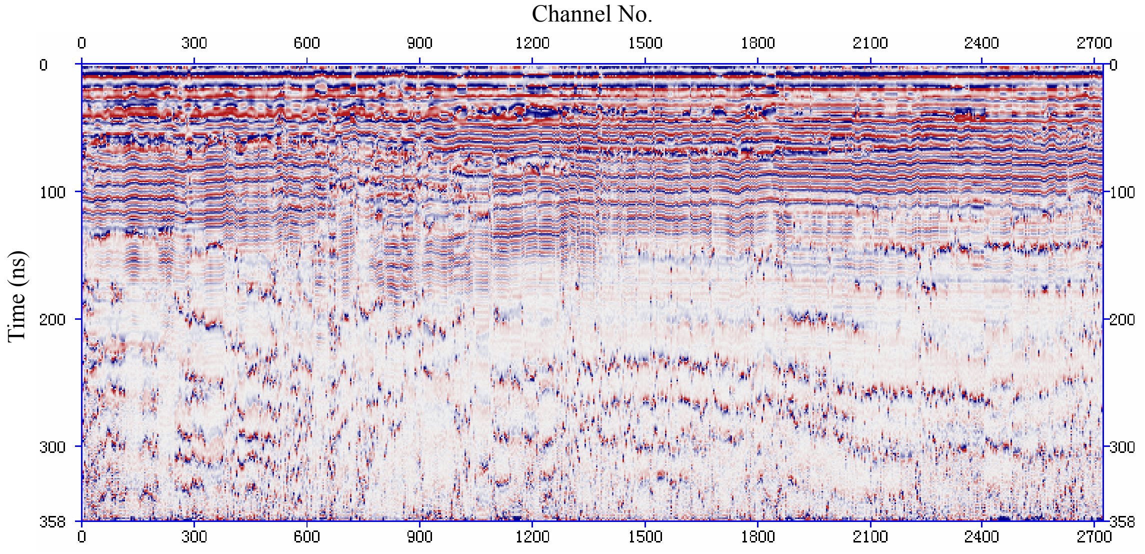

Fig.4 SIF attribute

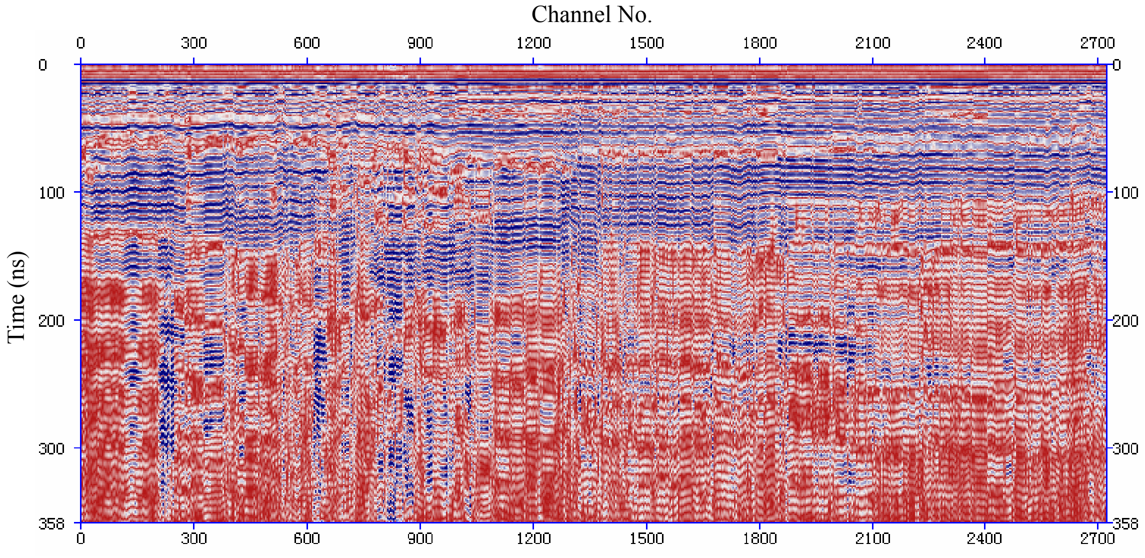

Fig.5 PIACIP attribute

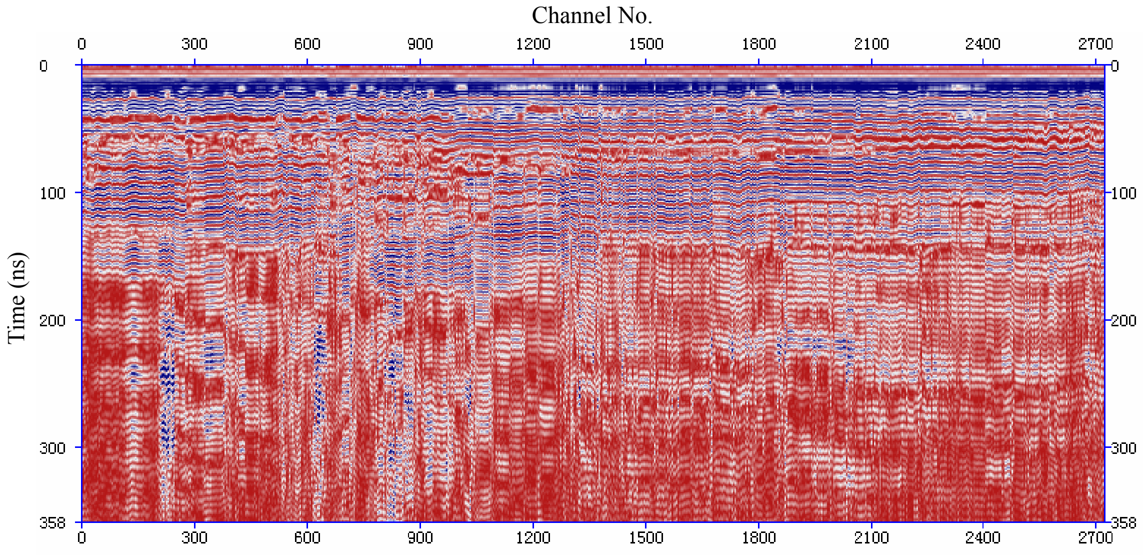

Fig.6 AL attribute
